# A Multidisciplinary Educational Approach for Children With Chronic Illness: An Intervention Case Study

**DOI:** 10.5334/cie.2

**Published:** 2020-01-09

**Authors:** Christopher Harden, Hannah Rea, Iris Buchanan-Perry, Beatrice Gee, Alcuin Johnson

**Affiliations:** 1 Children’s Healthcare of Atlanta, US; 2 University of Georgia, US; 3 Morehouse School of Medicine, US; 4 Emory School of Medicine, US

**Keywords:** Opportunity gap, Chronic illness, Sickle Cell Disease, Collaborative education, Community tutoring

## Abstract

Chronic illness requires frequent medical treatments and lifestyle restrictions that increase academic and socioemotional stressors for families. This paper presents academic intervention recommendations based on a hospital’s approach to improving educational outcomes for children with chronic illness. A case study on an intervention for a girl with sickle cell disease (SCD) and a history of stroke. SCD is a relatively common chronic illness that has physical and psychosocial side effects that are central to other chronic illnesses (Platt, Eckman, & Hsu, 2016). A quality improvement approach resulted in five cycles of interventions that were assessed with both qualitative and quantitative measures. The initial strategy of improving academics through collaboration among the school, hospital, and family resulted in psychosocial, but not academic, improvements. Frequent tutoring, which was most achievable using online platforms, resulted in the greatest gains. The girl passed previously failed classes and advanced to the next grade. Recommendations for how to improve academic outcomes for children with chronic illness using the presented intervention strategies are discussed.

School participation is foundational for the academic, social, behavioral, and emotional development of children and youth (Flook, Repetti, & Ullman, 2005). Therefore, it is vital to create effective interventions that mitigate barriers to schooling for vulnerable populations (Byrnes, 2008; Thompson, Lazarus, Clapper, & Thurlow, 2006). One such population is children with chronic illness for whom medical complications and treatments can create myriad academic and psychosocial challenges. As improved medical care rapidly increases the number of children living with chronic illness, and, therefore, attending school, interventions are needed in the United States of America (USA) that address these children’s multi-faceted barriers to schooling and development (Data Resource Center on Child and Adolescent Health, 2016–2017; Piel, Hay, Gupta, Weatherall, & Williams, 2013; Van Cleave, Gortmaker, & Perrin, 2010). Thus, the current study aimed to provide intervention recommendations based on a case study analysis of a multidisciplinary educational intervention for a student with the chronic illness sickle cell disease (SCD) living in the USA.

## Chronic Illness

Chronic illness is occasionally referred to using different terminology (e.g., special healthcare needs), and defining the concept is complex. Consistent with the literature in the medical field, the current paper defines chronic illness as any long-term or permanent medical condition that impacts everyday life (see review in Lubkin & Larsen, 2006; Thies, 1999). The complications associated with chronic illnesses accumulate and interact in a negative cycle that exacerbates both physical and socioemotional health (Kassim, Gladanci, Pruthi, & DeBaun, 2015). For example, both the symptoms of the disease and the treatments may fatigue the individual, which may result in further diminished attention, memory, and learning (Shaw & McCabe, 2008; Thies, 1999). Hospital visits, pain crises, and medication disrupt schooling, relationships, family dynamics, and financial security (DiNapoli & Murphy, 2002; Smith, Patterson, Szabo, Tarazi, & Barakat, 2013). These and similar factors, in turn, increase stress (American Academy of Pediatrics [AAP] American Public Health Association, & National Resource Center for Health and Safety in Child Care and Early Education, 2011; Tarnowski & Brown, 2000), which increases the risk for anxiety, depression, low self-esteem (Shaw & McCabe, 2008; Wodka & Barakat, 2007), and worsened physical problems both for the individual and for his/her caregivers (AAP Committee on School Health, 2000). Thus, interventions are essential for mitigating physical and psychosocial difficulties.

## Sickle Cell Disease

While all chronic illnesses have unique symptoms and stressors, SCD can serve as a model in research on children with chronic illness because it commonly co-occurs with a myriad of physical and psychological disabilities that characterize or are associated with other illnesses (Hassell, 2010). SCD is also important to study as it is the most common genetic disease worldwide (Centers for Disease Control and Prevention, 2017; Weatherall, Hofman, Rodgers, Ruffin, & Hrynkow, 2005). For example, it impacts about 10% of African Americans in the USA, and rates may increase as the proportion of African Americans in the population increases (Hugo, 2009; Sedrak & Kondamudi, 2019).

SCD is comprised of a group of genetic blood disorders in which blood or oxygen flow is blocked or stopped. Symptoms include severe pain, anemia, vision problems, jaundice, organ damage; in addition, 8–15% of children with SCD in the USA experience a stroke (Platt et al., 2016; Sedrak & Kondamudi, 2019). Strokes cause early death, increased risk for a second stroke, and lifelong problems in learning, executive functioning (i.e., attention, memory, impulsivity), language expression or understanding, neuro-motor skills (i.e., fine-motor tasks such as handwriting and gross-motor tasks such as balance and coordination), and overall cognitive development (de Montalembert & Wang, 2013; Ware & Helms, 2012). In sum, SCD has both acute and long-term effects on an individual’s physical, cognitive, and psychological well-being.

SCD-related physical symptoms may be managed by hospital visits and intensive medical care in the USA; however, that time in the hospital has consequences for academic achievement and daily functioning (AAP Committee on School Health, 2000). For example, the number of planned hospital visits for children with SCD in the USA ranges from twice per year to once every three weeks (Yawn et al., 2014). In addition, children with SCD have high rates of emergency room visits, hospital admissions, and sick days, all of which result in school absences and missed school instruction (Champaloux & Young, 2015).

## Academic Barriers for Children With Chronic Illness

Improved medical care has increased the lifespan for individuals with chronic illness, in general, and children with SCD, specifically, but for the growing number of children living with these conditions who are in need of school accommodations, improvements in school training or resources are still lacking in the USA. The No Child Left Behind Act (No Child Left Behind [NCLB] Act of 2001, 2002) was one attempt made by the USA Department of Education to improve academic progress through increased federal involvement in the education system. NCLB required schools to demonstrate adequate yearly progress (AYP), or achievement of projected growth. Some metrics of AYP included student attendance and standardized testing scores. Unfortunately, however, the NCLB standards negatively reinforced school systems for retaining students with chronic illness, as these were more likely to have poor attendance and related low-test scores (Irwin & Elam, 2011). While NCLB expired, the replacement, Every Student Succeeds Act (ESSA), continues many NCLB goals, including annual standardized testing for third and eighth grades. Under ESSA, states have more authority and flexibility over their education goals and consequences for under-performance, but similar standards and metrics of school accountability remain in use, and, thus, similarly have a negative impact on students with chronic illness (ESSA, 2015).

The Individuals with Disabilities Education Act (IDEA) of 2004 and the Rehabilitation Act (1973) were other federal efforts to increase equal opportunity for students with disabilities in the USA. IDEA (2004) required schools to provide individualized educational programming (IEP) for children with achievement-interfering medical conditions. The Rehabilitation Act (1973) prohibited schools from discriminating on the basis of disability. Again, however, this legislation fails to account for the unique sequelae of chronic illness (Shaw & McCabe, 2008). In sum, although the primary goals of NCLB (2002), ESSA (2015), IDEA (2004), and the Rehabilitation Act (1973) were to ensure equal educational opportunity for all children in a safe and inviting environment, children with chronic illness continue to be disadvantaged.

The limitations of USA education for children with chronic illness are repeatedly shown in qualitative and quantitative studies. Past research reported that educators are often unaware of or misunderstand the needs of students with chronic illness (Knauer, Baker, Hebbeler, & Davis-Alldritt, 2015; Olson, Seidler, Goodman, Gaelic, & Nordgren, 2004; Sexson & Madan-Swain, 1995; Shiu, 2001). As a result, many American schools treat children with chronic illness like other children who are truant from school by retaining them or holding them responsible for independently learning material missed during their absences (Balfanz & Byrnes, 2012; Irwin & Elam, 2011). This approach fails to account for the decreased energy and concentration that accompany the diseases and treatments, lifestyle restrictions, and other accumulating problems related to SCD, specifically, and chronic illness, generally (Magrab, 1985; Miller & Wood, 1991). Furthermore, as a result of feeling inadequately prepared, American teachers have reported feeling burdened by the student (Olson et al., 2004), the accommodations (Shiu, 2001), and by the expectations that the child will accomplish less as a result of his or her illness (Irwin & Elam, 2011; Sexson & Madan-Swain, 1995).

As students age, the organizational demands increase. In American secondary schools, students have more teachers, classes, and work. Thus, they grow further disadvantaged from the previous years of missed schooling and from their continuing psychosocial challenges (Balfanz & Byrnes, 2012). Overall, most of the aforementioned American academic policies have been shown to leave families and children with SCD and other chronic illnesses feeling abandoned and burdensome (Irwin & Elam, 2011). These negative experiences then add to challenges, behavioral problems, and risk of academic failure (Jimerson, Pletcher, Graydon, Schourr, Nickerson, & Kundert, 2006; Shiu, 2001; Thies, 1999).

In addition to the structural barriers to learning and classroom participation for patients with chronic illness, children with SCD and other chronic illnesses also face interrelated, interfering cognitive, psychological, and other deficits (Eiser, 1990; Nelms, 1989; Olson, Johansen, Powers, & Pope, 1994). For example, for children with or without SCD, stroke-related impairments in executive functioning can make it difficult to pay attention and to complete tasks (de Montalembert & Wang, 2013; Schatz & Roberts, 2007; Ware & Helms, 2012), which may increase the risk of peer rejection (Jellinek, Bostic, & Schlozman, 2007; Mukherjee, Lightfoot, & Sloper, 2000). Receptive and expressive language difficulties that occur with many illnesses, including stroke, may also negatively impact both learning and relationships (de Montalembert & Wang, 2013; Ware & Helms, 2012). Further, many children are aware of their academic and social shortcomings and may feel isolated, hopeless, and/or burdensome. Such sentiments predict psychopathology (e.g., anxiety), difficulty transitioning from hospital to school, decreased motivation, and academic failure (Irwin & Elam, 2011; Madan-Swain, Fredrick, & Wallander, 1999; Pinquart & Shen, 2011).

Finally, all of the physical and psychosocial difficulties often leave American families ill-equipped to help their child. Thus, families of children with chronic medical and behavioral problems expend increased resources to address their child’s many medical and psychological needs (Kuhlthau, Hill, Yucel, & Perrin, 2005). Not surprisingly, many parents experience stress, burden, and interferences in employment and finances related to their offspring’s deficits and increased daily care (Magrab, 1985; Stabile & Allin, 2012). Parents may experience guilt related to having passed on genetic diseases, realizations that the child’s life cannot be normal, and difficulty managing unpredictable episodes of pain (Magrab, 1985). Isolation from community and school resources, as well as negative past experiences with schools, have also been found to deplete trust and involvement in the school system (Knauer et al., 2015). Overall, the myriad stressors parents face may leave many without the ability to provide academic tutoring, emotional support, or school coordination to bridge academic opportunity gaps (Blanchard, Gurka, & Blackman, 2006).

## Academic Recommendations for Children With Chronic Illness

In recognition of the ongoing academic difficulties of children with chronic illness that are not addressed by NCLB (2002), ESSA (2015), IDEA (2004), or the Rehabilitation Act (1973), the American Academy of Pediatrics (AAP) made some recommendations for families, hospitals, and schools. For example, the AAP proposed that hospitals hire teachers to maintain a schooling routine during absences as well as collaboration with the school. Online schooling was also recommended to help with missed instruction (AAP Committee on School Health, 2000).

However, few academic interventions for children with chronic illness have been empirically studied or well defined. For example, the role of hospital teachers is not clearly defined, nor is it consistent across hospitals (Steinke, Elam, Irwin, Sexton, & McGraw, 2016). Most American programs are still focused on the hospital-to-school transition for children who were hospitalized for long periods of time, despite the fact that hospital stays are growing shorter (Shaw & McCabe, 2008). One randomized controlled trial analyzed the efficacy of a weekend workshop aimed at educating families on school accommodations for their child with SCD. However, the workshop was not found to improve academic outcomes compared to the control condition (Daniel et al., 2015).

AAP recommendations for collaborations and online schooling may help many families, but research is needed on the implementation and outcome of these strategies. First, online programs require motivation, independence, technical skills, access, self-discipline, time management, and self-esteem, all of which are likely to be challenges for children with chronic illness (Fernandez, Ferdig, Thompson, Schottke, & Black, 2016). Second, the feasibility and effectiveness of online programs for children with chronic illness require careful study because, compared to traditional schools, such programs are often shorter in duration, implemented by less qualified teachers (Irwin & Elam, 2011), unable to provide social development or community integration (AAP Committee on School Health, 2000; Gjengendal, Rostøen, Wahl, & Hanestad, 2003), and may pose economic and time burdens for parents (Macciomei & Ruben, 1989; North Carolina Department of Public Instruction. Exceptional Children’s Division, 2000). Finally, research is needed on best practices for and outcomes of the AAP recommendations for collaborations among schools, hospitals, and families in the USA (Irwin & Elam, 2011; Steinke et al., 2016). In particular, evidence-based practices are needed to help parents overcome the numerous personal and environmental barriers they face while trying to obtain school accommodations for their children (Bacon & Causton-Theoharis, 2013).

## The Present Study

Given the gaps in the literature and the need for educational programs, the following case study aimed at developing and evaluating an academic intervention that may be useful for children with chronic illness. Maya, a young girl with SCD and a history of stroke from a low-socioeconomic-status family in the USA, is presented as a case example because the intervention was developed with her and because the constellation of difficulties associated with SCD may serve as a model for chronic illness. However, the intervention has also been successfully used with children with other chronic illnesses in the hospital, so recommendations for other illnesses are considered.

Our initial hypothesis was that a systemic intervention with the parent, student, hospital, and school would improve Maya’s academic performance. Specifically, we expected that frequent collaborative meetings among these stakeholders would improve parent and child advocacy and increase school and hospital knowledge of respective systems, thereby leading to improved academic performance. We hypothesized that individual and group meetings with Maya’s mother would give her the self-efficacy, knowledge, and skills to advocate for and help her daughter. We expected that creating an environment that made Maya, her family, and her school feel safe, welcomed, respected, and engaged would bridge the learning and social gaps that resulted from her chronic illness symptoms and school absences.

## Methodological Framework

The study employed a quality improvement process that is typically implemented in health care delivery systems (Varkey, Reller, & Resar, 2007). The Plan-Do-Study-Act (PDSA) cycle entails effecting change by first planning an intervention, implementing the plan, studying the results, and acting on what is learned. The cycle of monitoring and modifying the system continues until the desired outcome is achieved.

Consistent with the quality improvement approach, goals were set (i.e., Maya will complete all requirements for promotion to the next grade). Next, stakeholders were determined: the student, caregivers, school personnel (teachers, case managers, guidance counselors), hospital personnel (teacher, medical providers, psychologist, social workers, nurses). Measures of goal achievement were selected (i.e., grades, state assessments, school attendance, parental attendance at school meetings, student preparedness for instruction, qualitative reports of academic and psychosocial progress). Finally, the plan was implemented in a real-world setting.

The quality improvement approach was first used with Maya, as the beginning of a larger effort to develop an academic intervention for children with chronic illness. We present Maya as a single case example because case study designs are rigorous ways to conduct repeated measures assessments with high internal validity. Thus, we believe this format best demonstrates the direct impact of the intervention on outcomes, controlling for confounding variables (Lobo, Moeyaert, Cunha, & Babik, 2017).

## Context

Maya was an 11-year-old African-American female entering the fifth grade at the start of the academic intervention (August 2015). She was diagnosed with sickle cell anemia and asthma at birth. Prior to the current intervention, she had multiple hospitalizations and emergency room visits for SCD and other related complaints (e.g., urinary tract infection, seizure). In preschool (October 2009), she presented with an acute cerebrovascular accident, which damaged the left and frontal lobes of her brain. In a third-grade neurocognitive exam, the neuropsychologist on our hospital team reported that Maya demonstrated normal functioning in intelligence, memory, visual learning and understanding, math, and socioemotional functioning. However, she exhibited abnormalities in adaptive functioning, attention/executive functioning, list learning, language, sensorimotor skills, reading, and written language. These neurocognitive issues placed her at risk for academic difficulties.

Prior to and during the academic intervention, Maya attended a public school in an economically disadvantaged, urban community. Beginning in second grade, Maya was classified as a student with a disability under IDEA. Thus, her school had to create an individualized education program (IEP) to facilitate her acquisition of government standards and achievement of personal goals (IDEA, 2004). Specifically, Maya’s IEP was supposed to provide her with extra time on assessments, check-ins with teachers to ensure understanding, shorter work intervals, concise directions, peer tutoring, and preferential seating. Extra time and preferential seating were supposed to be used for standardized assessments, not classroom tests. However, many of Maya’s specified accommodations, such as shorter work intervals and peer tutoring, were not implemented (e.g., Maya was given the same assignments and due dates as her classmates).

At the start of the intervention, Maya was at risk of failing the standardized assessments required by ESSA (2015) for students to advance to the next grade. As a resident of the state of Georgia, Maya was expected to take the Georgia Milestones Assessment (Georgia Department of Education, 2017) in grades three through eight. Maya was also required to pass math, reading, and two more of her five classes with a grade of 70 or above to advance grades (Georgia Department of Education, 2017).

Regarding family life, Maya lived with her mother. Maya’s mother did not respond to invitations to attend IEP meetings. Maya appeared disengaged from her education, particularly in the hospital. For example, she never responded to hospital lessons with work materials because she reportedly believed she was “not in school” and the teacher was “not [her] real teacher.”

The hospital staff (teacher, medical providers, psychologist, social workers, nurses) planned to foster collaboration among themselves, her mother, and her school to improve her school functioning. The hospital team reviewed her grades after every quarter. Changes were made to the intervention based on her performance. Below are descriptions of the outcome measures and interventions.

## Methods and Results

### Measures

#### Grades

The hospital team examined Maya’s grade reports quarterly. Her grades were the primary reason for modifications to the interventions.

#### State assessments

The Georgia Milestones Assessment System (Georgia Department of Education, 2017) is the ESSA (2015)-required standardized assessment in the state of Georgia. Assessments are given in math, reading, language arts, science, and social studies. Beginning in fifth grade, students do not advance grades if they fail math or reading exams. Based on exam performance, students are classified into four groups, as follows. “Beginning Learners” do not demonstrate proficiency and needed significant academic help. “Developing Learners” demonstrate some proficiency but still need academic support. “Proficient Learners” are prepared for the next grade. Finally, “Distinguished Learners” show advanced knowledge that makes them well prepared for school (Georgia Department of Education, 2017).

#### School attendance

The hospital team reviewed school attendance quarterly. The goal was to reduce the number of school days missed because absences contribute to poorer performance.

#### Parental attendance at school meetings and self-reported experience

Maya’s mother’s attendance at IEP and school meetings served as a measure of involvement at school. Parent reports of confidence, preparedness, and satisfaction with the intervention were obtained via interviews with the hospital staff.

#### Student preparedness for instruction and self-reported experience

Student preparedness and readiness for instruction were assessed during hospital instruction and school meetings. These factors were defined as Maya’s independent provision of books, materials, and computer, her self-report of book use and knowledge of assignments, as well as her self-report about her experiences during conversations with the hospital staff.

Below, please find a description of the five cycles of the intervention program.

### Cycle 1

#### Methods

The initial plan was to increase collaboration among and between hospital, school, and family while supplementing missed instruction with hospital tutoring. The collaboration had three purposes, to increase (a) the school’s understanding of Maya’s medical condition, (b) the hospital teacher’s knowledge of Maya’s academic requirements, and (c) relationships among parties to allow for continued improvements. The effort began at the start of Maya’s fifth-grade year when the hospital team and Maya’s mother sent a letter to Maya’s teachers, case manager, and school officials describing her symptoms, the rationale for hospital visits every three weeks, and the need for water and related bathroom breaks throughout the day because hydration decreases SCD pain. The letter also instructed teachers to inform substitutes about health-related accommodations in their lesson plans.

Similar information was reiterated in a school meeting with the hospital team, Maya’s mother, and the school team before the school year started. The in-person meeting allowed for rapport building as well as for further specification of SCD, Maya’s cognitive functioning after the stroke, best practices for children with chronic illness, and ways to improve and enhance Maya’s academic achievement. The hospital team focused on the importance of checking work, encouraging Maya to think aloud, checking for understanding by asking Maya to repeat instructions, providing additional work, and breaking down assignments into more manageable parts to minimize stress, confusion, and frustration.

With Maya and her mother, the hospital team implemented check-ins to increase and maintain buy-in, as well as to monitor the family’s psychosocial needs. For example, Maya and her mother initially expressed doubt that Maya’s performance could improve. Thus, the hospital team discussed with them the impact of effort on academic performance. The hospital team also helped the family create a homework schedule. The family was expected to complete homework and reading at the same time of day, at least five to six days per week. Maya and her mother were taught good work habits, including turning off electronics during study hours and posting the work schedule in a central location. For the first six weeks, hospital staff phoned and emailed Maya’s mother weekly to ensure that the schedule was followed. Subsequently, the hospital staff, Maya’s family, and the school counselor had a conference call every three weeks to follow-up on homework completion.

The hospital psychosocial team started a monthly parent support group, which we hoped would reinforce and develop Maya’s mother’s advocacy skills. Parents were provided with psychoeducation on agendas for school conferences, the process and purpose of an IEP, parental rights at IEP meetings, methods for increasing parental involvement at school, and ways to reduce bullying. Other topics were addressed per parent request. Parents also shared with each other to foster a positive, helpful group dynamic.

Finally, Maya received individualized instruction from the hospital school teacher during her one- to two-hour transfusion visits. The hospital-based learning session started with 20 minutes of reading. Maya then had to summarize the reading and give a prediction of what would happen next. She then discussed, organized, and completed other homework assignments with the hospital teacher.

#### Results

School collaboration, family support, and hospital instruction did not lead to the desired academic performance as Maya continued to fail math (62/F) and social studies (63/F) following cycle 1. At this time, she was passing science (84/B) and language arts (81/B).

An improvement was noted in parental participation at school. Maya’s mother began to attend all school meetings prepared and on time, even adjusting her work schedule to attend the meetings. Maya demonstrated more readiness for instruction in the hospital, as evidenced by her consistent provision of assignments and a laptop to the hospital teacher.

### Cycle 2

#### Methods

After completing Cycle 1, Maya continued to fail math and social studies. Therefore, the hospital team implemented strategies specific to those subjects for Cycle 2. Due to some perceived benefits from the first cycle, Cycle 2 included all of the Cycle 1 strategies, but with the addition of a computerized online program in math and social studies (Ixl.com, 2019) and a monthly group tutoring session with math and science specialists.

The computer program provided instruction and activities to children in elementary school. Maya could use the program at home, during clinic visits, and during breaks from school. The hospital staff could monitor the time spent on the program and academic progress through the website.

The hospital teacher also began a collaboration with local tutoring volunteers from the Georgia Institute of Technology (Georgia Tech) biomedical engineering (BME) undergraduate program, who provided free individual and group math and science tutoring. The approach to tutoring was an evidence-based personalized learning approach, in which instruction was tailored to address the needs and goals of individual students (Pane, Steiner, Baird, Hamilton, & Pane, 2017). The goal was to emphasize analysis and synthesis to improve cognitive complexity. As such, Maya was presented with final products at the beginning of each lesson (Lundstrom, Diekema, Leary, & Haderlie, 2015). She was then asked different types of questions (e.g., What do you see? Do you see what I am showing you?); she took notes on her observations, and broke down the final product into its component parts. For example, in a math lesson on the order of operations, Maya and the tutors read the problem out loud and talked through each step to learn the topic. Tutors also used creative means of teaching, such as playing a game where Maya was able to explore, feel, and visualize relationships among numbers. The hospital team regularly discussed Maya’s strengths, weaknesses, and progress with tutors in order to better monitor her progress and inform future intervention modifications.

#### Results

After finishing Cycle 2, Maya’s mathematics grade improved slightly, but she continued to fail the subject (68/F). The scaffolding and gentle feedback during tutoring improved Maya’s attitude toward learning, such that she reported feeling more comfortable about making mistakes and being better able to problem-solve to the right answer. Thus, we expected to see continued improvements from tutoring over time. Her social studies grade increased to a 78 from a 63, and she was now passing all of her other classes. Maya’s mother continued to attend school meetings and to implement the homework schedule.

### Cycle 3

#### Methods

During Cycle 3, all of the strategies implemented in Cycles 1 and 2 were continued as they had resulted in moderate improvements, but the method of instruction used by the BME tutors was revised to be more customized and accessible. Tutors met with Maya individually and focused on the school standards that the hospital teacher obtained from the school. The frequency of tutoring was increased to once per week over Skype.

Regarding Maya’s metacognitive and socioemotional skills, the hospital and school teams began providing formative assessments without grades, clear and specific feedback, previews for future lessons with the guidance of a tutor, and guided reflections on accomplished tasks. It was hoped that these practices would increase her motivation and confidence, and improve her study habits. The school counselor and case manager also met with Maya twice a month to address emotional and social concerns (e.g., study habits, social skills). Finally, school personnel provided Maya with peer helpers to ease her transitions back to school.

#### Results

Maya was able to meet the desired outcome, as she was no longer failing mathematics (88/B). Maya’s teachers recognized her improvements by granting her the Student of the Month award for her academic and emotional gains. The reading teacher reported an improved reading lexile score from the previous year, but Maya was still a year below grade level. Unfortunately, Maya’s science grade dropped (from 84/B to a 77/C); however, she showed increased interest in science. For example, she and her classmates reported benefiting from her science fair project on SCD, as her project enhanced everyone’s understanding of the disease and her symptoms. Maya’s mother continued to show improvements in school participation. She independently scheduled a parent-teacher conference to discuss the next steps.

### Cycle 4

#### Methods

As in previous cycles, all of the earlier strategies were continued in Cycle 4. In addition, a science specialist from the nearby Fernbank Science Center implemented individualized instruction for free during transfusion appointments to address Maya’s decreased science grade. As before, the hospital school teacher obtained Maya’s learning standards, objectives, and specific assignments from the school program educator and teacher. The hospital teacher passed this information to the Fernbank tutor and returned Maya’s completed work to the school via email or fax so she could receive credit.

To establish these partnerships required extra work and efforts on behalf of the researchers. That is, we had to train volunteers on confidentiality requirements and had to obtain a release of information consent to communicate among stakeholders. Initially, we had difficulty coordinating the date and time between Maya’s appointments and the Fernbank Science Center teacher, but this was resolved with flexibility and communication by all parties.

#### Results

Maya continued to meet the desired outcome in mathematics (89/B), but her science grade continued to fall (71/C). However, in conduct and work habits (e.g., working well within groups, staying on task, making wise use of class time), she progressed from “satisfactory” to “excellent” on her report card. Maya’s mother’s school involvement continued.

### Cycle 5

#### Methods

The strategies from the previous cycles were continued during Cycle 5. A Georgia Tech BME tutoring session in math and reading was added in preparation for the Georgia Milestones. Thus, Maya’s tutoring sessions increased from once to twice per week for one-hour sessions. All BME tutoring sessions were conducted via Skype. Due to her dropping science grade, we increased the frequency of her work with the Fernbank Science Center.

#### Results

Maya demonstrated growth on the state assessment. For the first time, she was now at the Beginning Learner level in math and reading, with scores of 490 and 521, respectively. These scores placed her in the 36^th^ percentile for math and 32^nd^ for reading. Her math score was lower than the mean score in the school (502) and state (510), but her reading score was about 15 points higher than the average score in the school and state. Maya progressed to the Developing Learner level in science and social studies. She was also passing all her classes and advanced to the next grade. A neurocognitive exam following the intervention (2017) indicated normal performance in most domains, including those that were previously weaknesses. Maya’s only remaining areas of normative weakness were in new learning and right-side motor skills.

Maya’s mother remained involved and continued the homework schedule. A review of the number of absent days at the end of the year demonstrated that, while Maya missed some school time for medical appointments, the hospital met its goal of less than 10% school days spent absent. This was achieved by adjusting some medical appointments to take place after school hours or on holidays.

## Discussion

The goal of the current study was to develop academic intervention recommendations for children with chronic illness based on a quality improvement case study conducted in the USA. Maya was presented as a case example because she, like many children with chronic illness in the country, experienced health-related school absences, physical impairment, and psychosocial stressors that led to academic failure (Haas & Fosse, 2008). Our intervention began by testing AAP recommendations that a hospital, school, family collaboration would boost skills and morale to increase academic achievement for children with chronic illness (AAP Committee on School Health, 2000; Olson et al., 2004). Additional interventions were introduced and evaluated systematically, based on Maya’s changes in performance.

The first cycle of the intervention demonstrated that the hospital staff’s involvement in IEP meetings helped foster collaboration among stakeholders. Consistent with our expectations, an increase in the hospital and family’s involvement in planning meetings improved the implementation and effectiveness of Maya’s IEP. For example, psychoeducation on Maya’s chronic illness and regular contact among parties increased the transmission of missed work and information on school standards. As a result, Maya’s hospital work became more productive, she received school credit for outside work, and she gained consistency across domains. The full team was also better able to adjust IEP goals and interventions using the diverse perspectives on Maya’s cognitive and academic strengths and weaknesses that each team member contributed.

Another anticipated product of the intervention was that Maya’s mother learned to independently advocate for and help her daughter. First, the hospital team’s modeling, as well as their assessment of the mother’s feelings of preparation, understanding of the language, and ability to articulate her daughter’s academic and social needs during IEP meetings, led to increased school involvement and advocacy. Maya’s mother’s confidence and skills were also bolstered through a parent support group. Parent support groups have many benefits, including offering an efficient method for disseminating information while providing social support that may mitigate stress (Minjarez, Williams, Mercier, & Hardan, 2011). Specifically, our group was consistent with and further supports previous research that parental advocacy interventions should focus on three goals: improving the parent’s ability to advocate, increasing the parent’s feelings of expertise, and fostering self-efficacy in the parent’s ability to protect the child (Lutenbacher, Karp, Ajero, Howe, & Williams, 2005).

While the current study did find some benefits to and effective methods for fostering a partnership among and between the family, school, and hospital, the collaboration did not result in the desired academic impact. Maya’s academic progress was minimal after Cycle 1, even with increased family and systemic support. Our findings contrast with those of previous research demonstrating that supportive, organized homes protect against academic failure for children with neuropsychological deficits (Fastenau, Shen, Dunn, Perkins, Hermann, & Austin, 2004) and other underperforming children in the USA (Berger, 1991).

Our case study also did not support recent recommendations that increased collaboration among stakeholders decreases the achievement gap for children with chronic illness, at least in USA institutions (AAP, American Public Health Association, & National Resource Center for Health and Safety in Child Care and Early Education, 2011). In sum, we suspect that increased support in the home environment and improved relationships among families, schools, and hospitals may have collateral benefits but may be insufficient for improving academic outcomes for children with chronic illness in the USA.

In response to Maya’s limited academic progress in Cycle 1, the hospital team explored alternative teaching approaches to make up for missed lessons in subsequent cycles. First, we supplemented tutoring with the hospital teacher with both an online program and in-vivo tutoring with local experts. When these latter approaches were found to yield only small improvements, the hospital team adjusted the frequency and format of tutoring in the following cycles until Maya was passing all classes. Given that Maya showed the most improvement when individualized tutoring with specialists was supplied both in-person and online, we hypothesize that technology is a particularly feasible and effective way of supplying missed instruction for children with chronic illness.

Research on online schooling and blended learning (combinations of in-person and online schooling) is still at an emerging stage; however, such approaches are increasingly used with children with chronic illness (Coy, 2014; Stella & Corry, 2017). It has been suggested that children with chronic illness may benefit from the individualized pacing and freedom to make mistakes without peers around that is uniquely available with online formats (Stella & Corry, 2017). It is also expected that online schooling may promote metacognitive skills, such as autonomy and responsibility (Terras, Leggio, & Phillips, 2015). Some caveats are in order; however. Online schooling lacks peer interaction and requires some degree of technical skills, self-discipline, time management, self-esteem, responsibility, motivation, and economic resources (Fernandez et al., 2016).

Based on our case study, we expect that web programs with accountability components will be most helpful for students with chronic illness, negative academic histories, and co-occurring psychosocial stressors. For example, Maya’s grades improved slightly following the introduction of a web-based program when the hospital team could monitor the number of hours she spent on the application and progress on assessments, even though her reported motivation was low at the time. Additionally, Maya made the most gains when tutoring was offered via video chatting. We expect that online tutoring resulted in greater improvements than the more generic online program because the former included individualized learning and the human interaction had socioemotional benefits that appeared to bolster self-efficacy and motivation. Thus, our findings are consistent with previous literature that online programs are more effective when they maximized teacher-student interactions (Greer, Rowland, & Smith, 2014).

Regarding the feasibility of the intervention, the hospital team found many ways to reduce the cost of each intervention. Specifically, partnerships with universities, community organizations, the hospital volunteer coordinator, and the school team allowed for affordable educational opportunities. The hospital was able to subsidize the online program and to provide Maya with a computer in the hospital library, as needed. The hospital team’s consultation during IEP meetings also helped them secure additional interventions for Maya, such as meetings with school counselors. The current study suggested that capitalization of existing tutoring programs, knowledge of school requirements, and effective advocacy may allow other areas to similarly provide cost-effective interventions.

While many goals were met, there were challenges. First, Maya showed resistance at the beginning, likely because of her previous negative experiences in school. Addressing Maya’s self-confidence and approach to learning using strategies such as clear and specific feedback, extra time to learn material before it was introduced in class, and regular meetings with the school counselor likely increased buy-in. We conclude that hospital staff, family, and school should consistently assess and address psychosocial factors, including attitudes toward school and problem-solving abilities, particularly if self-directed activities like online schooling will be used.

The second challenge involved keeping the team focused and hopeful when strategies did not result in the desired improvements. Maya, her mother, the hospital team, and the school learned the value of perseverance and patience when Maya’s grades declined or did not appear to improve over the course of five cycles in two years. One helpful method of maintaining morale was for the school program educator to meet monthly with the hospital team to analyze data, discuss and monitor the process, and make adjustments.

The final challenge was the limited frequency of Maya’s hospital visits. It was difficult to supplement missed instruction only once every three weeks. Thus, frequent communication among all parties was essential to ensure that multiple workers in various domains could assist and provide expertise. The option to provide Maya’s hospital tutoring via online sessions was also important.

### Future Directions

Maya has made academic and social progress since the beginning of the intervention and since the study concluded. When she progressed to grade 6, she passed all of her classes each semester. She continued to pass the Georgia Milestones Assessment and all her classes through middle school and will begin high school in the fall. She also exhibited neurocognitive growth in domains that negatively impact academic performance directly following the intervention (e.g., executive functioning, language, reading, and writing). In looking across the cycles, it is advised that individualized, frequent tutoring occurs early in academic interventions. It is still recommended that interventions begin with a collaboration across the hospital, school, and family to improve the provision of subsequent interventions. Furthermore, collaboration among stakeholders may allow for continued analysis of grades and other outcome measures in order to guide intervention modifications.

Maya was the lead person in this inquiry. This inquiry exemplified the curiosity, inquiry skills, and scholarly competencies needed to investigate a situation that leads to overall positive attitude, confidence, and especially student achievement. The team took the time to review, interpret the findings, reflect, and adjust based on Maya’s data and her feedback in making decisions in Cycles 1–5. The process started slow, yet with the ongoing communication between family, school, and hospital and effective community interactions throughout this process, Maya benefited. Maya helped the team developed some best practices in working with children with a chronic illness facing adverse life circumstances.

In reviewing the cycles and sharing these findings, the hospital team hopes to implement and research similar academic interventions that include collaborations among the family, hospital, and school as well as in-person and online tutoring for children with SCD and other chronic illnesses (e.g., traumatic brain injury, stroke, and autism spectrum disorder). The hospital staff is beginning these efforts by working with Maya’s school district’s special education coordinator in order to collaboratively disseminate the intervention to other children. We are also evaluating electronic networks (e.g., video conferencing, chat rooms, email) that may maintain peer interactions in academic experiences that occur during medical appointments. We further hope to cover more academic subjects (e.g., literacy) through collaborations with other community organizations, while retaining our partnerships with Georgia Tech and the Fernbank Science Center. Finally, the hospital team is trying to include summer interventions to continue to bridge the gap for children with chronic illness.

Children like Maya fight to live and survive their medical challenges; therefore, it is vital we give them every opportunity to complete their education and allow them to reach their fullest potential.
